# Indoxyl Sulfate Elimination in Renal Replacement Therapy: Influence of Citrate- versus Acetate-Buffering Component during Bicarbonate Dialysis

**DOI:** 10.1155/2018/3985861

**Published:** 2018-08-15

**Authors:** Radomír Hyšpler, Alena Tichá, Roman Šafránek, Petr Moučka, Zora Nývltová, Karolína Štochlová, Sylvie Dusilová-Sulková, Zdeněk Zadák

**Affiliations:** ^1^Department of Clinical Biochemistry, Faculty of Medicine in Hradec Kralove, University Hospital Hradec Kralove and Charles University, Hradec Kralove, Czech Republic; ^2^Hemodialysis Centre, Faculty of Medicine in Hradec Kralove, University Hospital Hradec Kralove and Charles University, Hradec Kralove, Czech Republic; ^3^Vyzkumny Ustav Organickych Syntez A.S. (VUOS-Analytika), Rybitvi, Czech Republic

## Abstract

Indoxyl sulfate has been identified as a major factor in the dysregulation of several genes. It is classified as a poorly dialyzable uremic toxin and thus a leading cause in the poor survival rate of dialysis patients. A monocentric, prospective, open cohort study was performed in 43 male patients undergoing chronic renal replacement therapy in a single hemodialysis center. The aim of the study was to determine the influence of acetate- versus citrate-buffered dialysis fluids in hemodialysis (HD) and postdilution hemodiafiltration (HDF) settings on the elimination of indoxyl sulfate. Also, additional factors potentially influencing the serum concentration of indoxyl sulfate were evaluated. For this purpose, the predialysis and postdialysis concentration ratio of indoxyl sulfate and total protein was determined. The difference was of 1.15 (0.61; 2.10), 0.89 (0.53; 1.66), 0.32 (0.07; 0.63), and 0.44 (0.27; 0.77) *μ*mol/g in acetate HD and HDF and citrate HD and HDF, respectively. Acetate HD and HDF were superior when concerning IS elimination when compared to citrate HD and HDF. Moreover, residual diuresis was determined as the only predictor of lower indoxyl sulfate concentration, suggesting that it should be preserved as long as possible. This trial is registered with EU PAS Register of Studies *EUPAS23714*.

## 1. Introduction

Indoxyl sulfate (IS) is an important uremic toxin; therefore, its levels in plasma have been extensively studied in end-stage renal disease [1]. Originating as indole, which is the result from tryptophan degradation by large bowel bacteria, it is conjugated with sulfate in the liver [1]; however, vegetarian patients often show lower IS concentrations, probably due to lower residual protein present in the colon and the difference in bowel microbiota [2]. Further, the concentration in plasma of IS correlates with protein intake even in subjects with normal kidney function [3], although patients with chronic kidney disease show reduced IS serum levels by 37% when following a low protein diet [4].

Indoxyl sulfate is a small solute (MW = 213 g/mol) that is in >90% reversibly bound to plasma proteins, predominantly to albumin [5]; thus, tubular secretion is the major elimination pathway [1]. However, dialytic clearance is low and dialysis patients show 10–20 times higher concentration of IS when compared to normal values. The level of IS in plasma is a significant predictor of major cardiac events [6], although other authors have found no association at all [7]. Regardless, a recent study identified IS as a major factor in the dysregulation of almost 2000 genes *in vitro* [8]. Moreover, poorly dialyzable uremic toxins are the leading cause of poor survival in dialysis patients. Therefore, an improved elimination by dialysis is needed.

In this regard, increased dialysis time has not been effective [9]. Further, dialyzer flow rate and surface area have little impact on protein-bound toxin elimination [10]. It must be mentioned that the methods in these two studies were based on diffusion without any significant convection. Moreover, adding convection to hemodialysis (such as in hemodiafiltration) was found to have negligible effects on toxin removal [11] in a single dialysis session, although others report a significant reduction of ~15–20% over a study period of six months [12].

IS binding to albumin is driven by electrostatic and *van der Waals* forces; therefore, its dissociation constant is dependent upon ionic strength (*μ*), dilution, and pH [13, 14]. Traditionally, sodium acetate has been used as a buffer to adjust the pH of the dialysate solution. Quite recently, sodium citrate was introduced into renal replacement therapy solutions in an attempt to reduce clot formation on the dialysis membrane and treatment-induced inflammation [15, 16]. Because the ionic strength of a solution is measured by the total ion concentration multiplied by their squared charge, polyvalent ions (citrate) increase the ionic strength of a dialysate while the concentration of other solutes remains the same. The difference in ionic strength of a citrate solution (0.8 mmol/l sodium citrate and 0.3 mmol/l sodium acetate) is 2.1 mmol/l higher than an acetate solution (3 mmol/l sodium acetate). This difference is relatively small; however, it could be significant due to the repeated passage of the patient's blood through the dialyzer cartridge and the large amount of dialysate used during a session of renal replacement therapy (RTT) (about 120 liters). Having stated that the total ion difference of both fluids is negligible (about 0.3 mekv/l), the citrate solution has a different metabolic behavior resulting in postdialysis pH and base excess increments regardless [15].

Moreover, citrate anions bind to albumin [17] and its supraphysiological concentration (>150 *μ*mol/l) is able to shift the albumin equilibrium towards a B conformation [18]. This could change the interaction between binding sites I and II, diminishing the level of free, dialyzable uremic toxins [19]. Thus, the behavior of citrate *in vivo* is too complex to be extrapolated from *in vitro* experiments and a clinical study is therefore necessary.

The aim of this study was to determine the influence of acetate- versus citrate-buffered dialysis fluids in hemodialysis (HD) and postdilution hemodiafiltration (HDF) settings on IS elimination considering, in addition, other factors potentially influencing the serum concentration of IS.

## 2. Methods

### 2.1. Patients and Study Design

A monocentric, prospective, open cohort study was performed in 43 male patients from a single hemodialysis center undergoing chronic RRT. All patients involved were under a maintenance dialysis regime for at least three months. The study focused primarily on assessing the serum concentration of IS, predialysis and postdialysis, and its relation to the dialysate-buffering component (acetate versus citrate) in two different modalities of hemodialysis—low-flux hemodialysis and high-flux hemodiafiltration. The RRT procedures used in this study are named ACET-HD, CIT-HD, ACET-HDF, and CIT-HDF, respectively.

All procedures were approved by the Ethics Committee (Ref. number 2015 06 S25P) according to the Declaration of Helsinki (June 1964 and later amended). The included patients were properly informed of the study's aim and methods in both verbal and written form, and an informed consent was obtained for every dialysis protocol relevant to this study. The inclusion criteria encompassed maintenance dialysis for at least three months, willing and capable to cooperate with the procedures, clinical stability for at least a month before the start of the study, intradialytic hemodynamic stability, and outpatient basis of the treatment. The exclusion criteria included acute inflammation, malfunction of vascular access for dialysis, significant comorbidity such as cardiac failure, severe hepatopathy, and extrarenal complications of diabetes mellitus. This trial is registered with EU PAS Register of Studies *EUPAS23714.*

### 2.2. Dialysis Solutions and Procedures

Two commercially available dialysis solutions were compared in this study (Table 1). The only difference was in the addition of acetate 3 mmol/l (ACET solution) or citrate 0.8 mmol/l, acetate 0.3 mmol/l (CIT solution). Both dialysis solutions contained identical ion and glucose concentrations, only the potassium concentration was adjusted in the range 2–4 mmol/l, according to serum concentrations.

Two RRT modalities were tested: conventional low-flux hemodialysis (HD) and online high-flux postdilution hemodiafiltration (HDF). Polysulfone dialyzer membranes (surface area 1.8 m^2^) were used in both procedures, keeping a constant dialysate temperature of 36.0°C.

In HD mode, a low-flux dialyzer with an ultrafiltration coefficient of 14 ml/mmHg/h was used. In HDF mode, a high-flux dialyzer with an ultrafiltration coefficient of 99 ml/mmHg/h was used. Sieving coefficients of >0.8 and <0.001 were used for *β*_2_-microglobulin and albumin, respectively. Fluid substitution was applied in automatic mode to allow a high convective volume. HD and HDF parameters were kept constant during the study. Cumulative blood flow serving as an approximate to dialysis adequacy was recorded in each procedure and kept similar to ensure comparable efficacy of the evaluated procedures. Also, the Kt/V index was calculated according to the Daugirdas II equation. Body weight change, total ultrafiltration, and substitution volumes were recorded. All procedures lasted for 240 minutes.

### 2.3. Study Design

Prior to the study, the patients were dialyzed using HD or HDF following a physician's decision. Four RRT modalities were introduced into the patient's individual RRT schedule (ACET-HD, CIT-HD, ACET-HDF, and CIT-HDF), always as a first procedure in four consecutive weeks. The total study time was of 22 days, and the sequence of procedures was kept constant.

A randomized sequence was considered unnecessary because midweek dialysis sessions were different between individuals, that is, 10 patients were treated using ACET-HD, 4 using CIT-HD, 27 using ACET-HDF, and 2 using CIT-HDF procedures.

The study protocol also involved the collection of a blood sample (4 ml peripheral blood) immediately before RTT and prior to heparin administration, using a standardized “slow-flow” method at the end of RTT in accordance with KDOQI guidelines [20]. All samples were immediately processed in accordance with the institutional standards. More extensive tests (including albumin, sodium, potassium, magnesium, total calcium, and phosphate) were performed prior to the first experimental procedure.

### 2.4. Analytical Methods

Routine clinical chemistry parameters (urea, creatinine, total serum protein, albumin, sodium, potassium, magnesium, total calcium, and phosphate) were determined using an automated analytical system cobas 8000 (Roche AG, Basel, Switzerland). Citrate anions were determined using capillary electrophoresis PrinCE (Prince Technologies B.V., Emmen, The Netherlands) and indirect detection by spectrometry at 375 nm using sodium chromate (10 mmol/l) in tetradecyltrimethylammonium bromide buffer (0.46 mmol/l).

A simple analytical procedure was developed to determine IS potassium salt in blood serum by an ion-pairing ultrahigh performance liquid chromatography using an electrochemical detector (UltiMate 3000 Series, Thermo Fisher Scientific, Waltham, MA, USA). Blood samples were drawn into tubes containing a gel separator, allowed to clot for 30 min, centrifuged at 2000*g* for ten minutes, and serum aliquots were kept at −75°C until batch analysis.

Serum samples (350 *μ*l) were diluted with 350 *μ*l ultrapure water and vortex mixed with 700 *μ*l 7% perchloric acid to deproteinize samples and to release all bound IS into its free form [4]. After 5-minute incubation at room temperature, the samples were centrifuged for 10 minutes at 10,000*g*/4°C. The supernatant was transferred to the glass autosampler vial and 10 *μ*l injected into the liquid chromatograph.

The analyses were performed on a Kinetex XB-C18 (100 × 4.6 mm, 5 *μ*m) analytical column equipped with a guard column at a flow rate of 0.3 ml/min. The mobile phase was 85% phosphate buffer (0.025 mol/l, pH 4.2, containing sodium dihydrogen phosphate, sodium 1-octanesulfonate, and ethylenediaminetetraacetic acid) and 15% methanol. IS was eluted at 5.5 minutes in an isocratic run. The detector voltage was set at +400 mV ([21], with modifications). The quadratic calibration curve was prepared in a concentration range of 12–400 *μ*mol/l and used for quantitation. The ratio calculation of total serum protein was chosen as compensation for the distribution pool constriction after dialysis.

### 2.5. Statistics

All statistical analyses were performed using the SigmaStat software v3.1. (Systat Software Inc., US). The data is presented as median (interquartile range) or mean ± standard deviation. The statistical difference between the groups was tested using a Mann–Whitney rank-sum test, and *p* ≤ 0.05 was considered statistically significant. The parameter's correlation was tested using a Spearman rank-order correlation test.

## 3. Results

### 3.1. Patient Data

Forty-three male patients aged 67.6 ± 11.5 years were included in the study. The underlying renal diseases found were polycystic kidney disease (*n* = 3), diabetic nephropathy (*n* = 18), glomerulonephritis (*n* = 11), interstitial nephritis (*n* = 9), kidney carcinoma (*n* = 1), and vasculitis (*n* = 1). The median duration of the dialysis treatment was of 4.0 years (interquartile range 2.65, 8.12, maximum 30.9 years). The mean ideal body weight was of 82.5 ± 17.1 kg, body mass index of 27.9 ± 4.88 kg/m^2^ and body surface area of 1.99 ± 0.2 m^2^. Anuria was present in 48.8% of the patients, whereas the remaining patients had residual diuresis of 1010 ± 410 ml/day.

### 3.2. Treatment Data and Dialysis Adequacy

The Kt/V index, according to the Daugirdas II equation, was of 1.36 (1.21; 1.50), and the equilibrated Kt/V index was 1.19 (1.06; 1.31). The Kt/V index was not statistically different among the evaluated RRT procedures. The treatment data (cumulative blood flow, total ultrafiltration, and substitution volumes) are shown in Table 2.

### 3.3. Laboratory Data

The predialysis concentration of IS was similar before each RRT procedure; however, its distribution was quite scattered within the study group. Regardless, the predialysis concentration of 24 patients (55.8%) remained in the same tertile throughout the study, whereas 39 patients (90.7%) were found within the same tertile in 3 out of 4 conducted evaluations, reflecting steady dietary habits and stable large bowel microbiome (Table 3).

**Figure 1:**
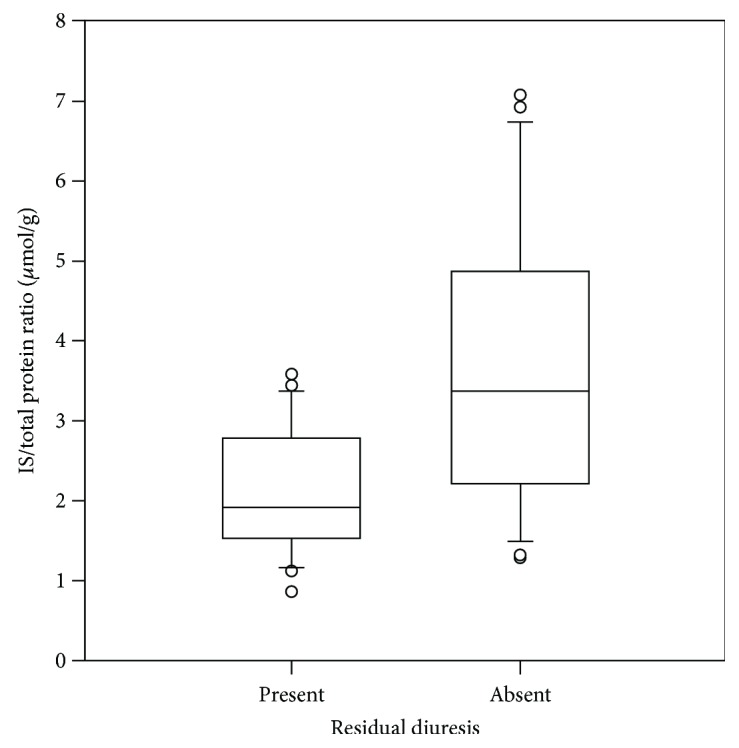
The predialysis ratio of IS/total protein in anuric patients and patients retaining residual diuresis (*p* = 0.002).

**Figure 2:**
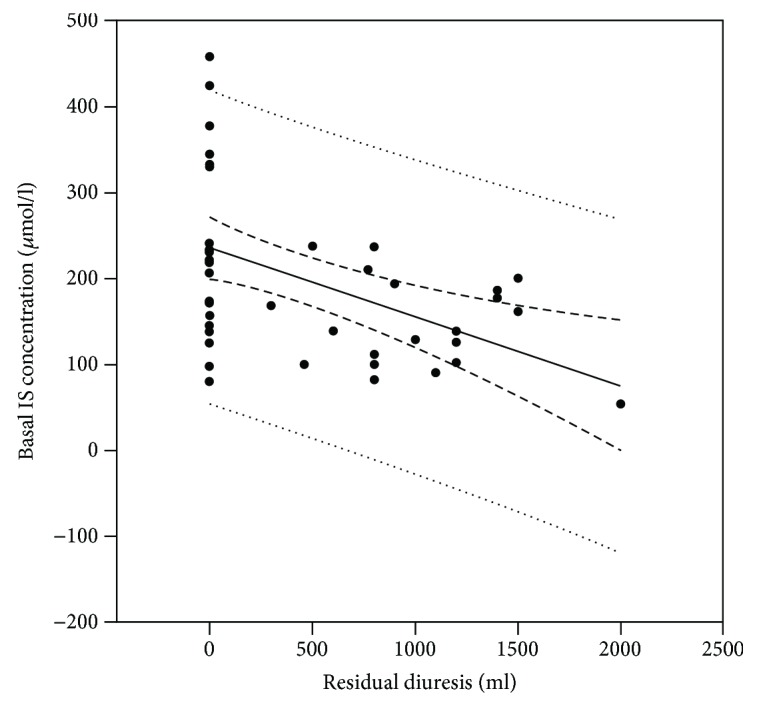
The correlation of basal (predialysis) concentrations of IS to residual diuresis volume in all patients involved in the study (*R* = −0.473, *p* = 0.001).

Further, residual diuresis was found as a positive influencing factor on the concentration of IS (Figures 1 and 2); therefore, this data was also analyzed separately in anuric and residual diuresis patients (Table 4).

**Figure 3:**
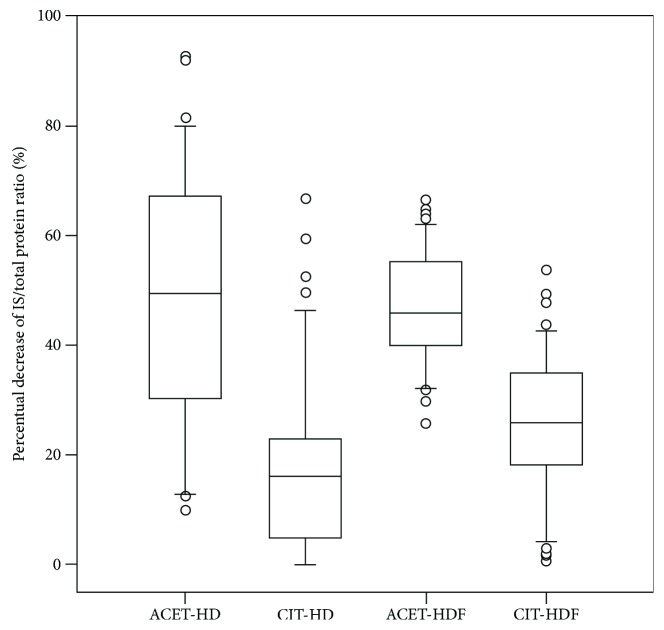
The comparison of percentual decrease of IS/total protein ratio in treatment modalities studied. The statistical difference between the groups is *p* < 0.001, except ACET-HD and ACET-HDF, where no difference was found.

When evaluating IS concentration during an RRT session, the acetate buffer hemodialysis modality was found to be superior to citrate buffer hemodialysis in decreasing its concentration (expressed as %) (Figure 3). Concerning the hemodiafiltration mode, the citrate buffer also performed worse when compared to the acetate solution. Furthermore, ACET solution was found to be better suited for hemodialysis or hemodiafiltration procedures when concerning IS removal using the abovementioned dialysis membranes.

Citrate serum concentrations after RRT were of 287 (249; 454) *μ*mol/in CIT-HD and 336 (282; 357) *μ*mol/in CIT-HDF versus 120 (27; 137) *μ*mol/in ACET-HD and ACET-HDF (*p* = 0.03).

Prior to the first experimental procedure, total protein in serum was of 68.3 (65.5; 71.5) g/l, albumin 40.2 (38.8; 42.5) g/l, sodium 139 (137; 141) mmol/l, potassium 5.3 (5.0; 5.7) mmol/l, magnesium 0.9 (0.84; 1.01) mmol/l, total calcium 2.17 (2.05; 2.29), and phosphate 1.74 (1.43; 2.14). No correlation between IS levels and these parameters was found before the first experimental procedure was administered, probably due to the inherent large scatter of IS concentration in RRT patients.

## 4. Discussion

The present study, although small in scale, evaluated the influence of these buffering components over IS elimination; to the best of our knowledge, such reports are yet unavailable elsewhere. The most frequently used RRT modalities, hemodialysis and hemodiafiltration, were used during our tests while trying not to disturb the patient's RRT steady state during their normal schedule. Due to the ultrafiltration during HD and HDF procedures, a significant constriction in the distribution pool could be observed. Thus, IS concentration to total protein ratio was calculated and used in the statistical evaluations.

When comparing HD and HDF in this study, the dialysis fluid containing acetate performed slightly better in hemodialysis without convection, contrary to previous trials which found negligible or only a slight improvement in hemodiafiltration. Anuric patients showed a significant improvement with the HD procedure, probably due to higher IS concentrations. This superiority was not observed in the residual diuresis group. In contrast to acetate, the fluid containing citrate was more efficient in hemodiafiltration, although only when expressed as a decrement (%) of the IS/total protein ratio. As mentioned in Introduction, “single session” studies are less sensitive to small changes in uremic toxin elimination, but long-term studies cannot distinguish between direct elimination and secondary metabolic effects of the RRT modality employed.

The primary goal of this study was to compare both dialysates. Surprisingly, both RRT modalities performed much worse when using a citrate dialysate compared to that containing acetate. Slightly higher ionic strength was probably outweighed by the changes in albumin conformation caused by citrate anions and reduction of the free, dialyzable fraction of IS in either the dialyzer cartridge or the intravascular pool. A previously described slight pH and base excess increment during citrate HD or HDF may further increase the fraction of B conformer albumin and intensify the effect of citrate anions. In this regard, the significant influence of minor buffering components in the dialysis fluids on IS clearance is observed in this study for the first time.

However, there was no difference in the Kt/V index according to the Daugirdas II equation, regardless of the cumulative blood flow intentionally kept constant in all procedures; therefore, the difference in IS elimination cannot be explained by the efficacy of the evaluated procedures. Further, several parameters were tested concerning their correlation with IS concentration; however, residual diuresis was the only significant factor found. Residual tubular secretion of IS results in its lower concentration in serum, which is beneficial for the patient; therefore, care should be taken in preserving residual diuresis as long as possible.

Protein-bound toxins have been identified as an important problem in recent years; therefore, it is necessary to determine their concentration by means of an inexpensive procedure, allowing us to determine the elimination efficacy of protein-bound solutes by RRT and to identify those patients with excessive toxin levels. Thus far, up-to-date dietary protein restriction is the most effective intervention, balancing protein intake to prevent malnutrition and to eliminate the excess of aromatic amino acids.

The results of this study draw attention to the influence of minor buffering components in dialysis solutions, providing an important and improved alternative to eliminate protein-bound toxins. Moreover, these results could direct the focus of research at the anionic components of dialysis solutions. However, this study was limited by its small-scale, single-session evaluation design and evaluation of only two types of dialyzer membranes. Also, the behavior of other protein-bound uremic toxins could be different and therefore needs to be assessed as well.

## 5. Conclusions

The elimination of protein-bound toxins by contemporary RRT techniques remains inadequate. The elevated concentrations involved are associated with higher oxidative stress, endothelial dysfunction, and increased risk of cardiovascular events. Acetate-buffered dialysis fluids performed significantly better in HD as well as HDF procedures when compared with citrate-buffered fluids. Further, residual diuresis is a major factor in eliminating IS. These changes in buffering components are quite interesting to say the least, although they are yet an underestimated possibility in improving the inadequate indoxyl sulfate elimination during renal replacement therapy.

## Figures and Tables

**Table 1:** Composition of dialysis solutions.

Component ions	ACET solution	CIT solution
Sodium (mmol/l)	138	138
Potassium (mmol/l)	2–4	2–4
Calcium (mmol/l)	1.25	1.25
Magnesium (mmol/l)	0.5	0.5
Chloride (mmol/l)	106–109	106–109
Acetate (mmol/l)	3	0.3
Citrate (mmol/l)	0	0.8
Bicarbonate^∗^ (mmol/l)	37	37
Glucose (mmol/l)	5.5	5.5

^∗^Concentration before reaction in mixture.

**Table 2:** RRT parameters.

Treatment modality	Q cumulative	Total ultrafiltration (ml)	Total substitution (l)
ACET-HD	70.9 (65.9; 82.8)	3350 (2525; 4000)	—
CIT-HD	68.0 (65.9; 82.4)	3350 (2325; 4000)	—
ACET-HDF	70.3 (62.8; 81.4)	3000 (2150; 3850)	21.1 (17.6; 24.5)
CIT-HDF	66.0 (59.9; 81.7)	3150 (2275; 3925)	20.7 (18.4; 23.0)

**Table 3:** IS concentrations and calculated values in the study group.

Treatment modality	Predialysis IS concentration (*μ*mol/l)	Postdialysis IS concentration (*μ*mol/l)	Predialysis IS/TPROT ratio (*μ*mol/g)	Postdialysis IS/TPROT ratio (*μ*mol/g)	Difference in IS/TPROT ratio (*μ*mol/g)	*p* values between RRT procedures
ACET-HD	175 (126; 237)	98.8 (52.8; 145)	2.55 (1.82; 3.46)	1.35 (0.67; 1.96)	1.15 (0.61; 2.10)	^a^ versus ^b^ < 0.001^c^0.018^d^ < 0.001
CIT-HD	143 (103; 212)	133 (101; 200)	2.30 (1.64; 3.23)	1.80 (1.32; 2.58	0.32 (0.07; 0.63)	^b^ versus ^c^ < 0.001^d^0.062 (n.s.)
ACET-HDF	157 (100; 227)	83.7 (56.2; 135)	2.28 (1.37; 3.26)	1.17 (0.78; 1.85)	0.89 (0.53; 1.66)	^c^ versus ^d^ < 0.001
CIT-HDF	135 (92.2; 179)	107 (73.8; 157)	2.10 (1.43; 2.70)	1.39 (0.99; 2.15)	0.44 (0.27; 0.77)	

The statistical difference between predialysis values was not found. ^a^ = ACET-HD; ^b^ = CIT-HD; ^c^ = ACET-HDF; ^d^ = CIT-HDF procedure.

**Table 4:** Calculated values in anuric patients and patients retaining residual diuresis.

Treatment modality	Difference in IS/TPROT ratio (*μ*mol/g)	*p* values between RRT procedures	Difference in IS/TPROT ratio (*μ*mol/g)	*p* values between RRT procedures
	In anuric patients		In patients retaining residual diuresis	
ACET-HD	1.70 (1.02; 2.58)	^a^ versus ^b^ < 0.001^c^0.041^d^ < 0.001	0.77 (0.43; 1.45)	^a^ versus ^b^0.002^c^ n.s.^d^0.002
CIT-HD	0.44 (0.20; 0.79)	^b^ versus ^c^ < 0.001^d^n.s.	0.18 (0.0; 0.36)	^b^ versus ^c^ < 0.001^d^n.s.
ACET-HDF	1.43 (0.75; 1.99)	^c^ versus ^d^ < 0.001	0.70 (0.44; 1.41)	^c^ versus ^d^ < 0.001
CIT-HDF	0.54 (0.27; 1.11)		0.37 (0.26; 0.72)	

^a^ = ACET-HD; ^b^ = CIT-HD; ^c^ = ACET-HDF; ^d^ = CIT-HDF.

## Data Availability

All relevant data are within the paper.
